# Iridescence and hydrophobicity have no clear delineation that explains flower petal micro-surface

**DOI:** 10.1038/s41598-020-67663-6

**Published:** 2020-06-30

**Authors:** Jair E. Garcia, Mani Shrestha, Laura Ospina-Rozo, Chaitali Dekiwadia, Matthew R. Field, Ji Sheng Ma, Nhiem Tran, Adrian G. Dyer, Kate Fox, Andrew D. Greentree

**Affiliations:** 1 Bio-Inspired Digital Sensing Solutions (BIDS) Lab, School of Media and Communication, RMIT University, Melbourne, 3001 Australia; 2 Faculty of Information Technology, Monash University, Clayton, 3800 Australia; 3 School of Biosciences, The University of Melbourne, Parkville, 3053 Australia; 4 RMIT Microscopy and Microanalysis Facility (RMMF), RMIT University, Melbourne, 3001 Australia; 5 Monash Centre for Electron Microscopy (MCEM), Monash University, Clayton, 3800 Australia; 6 Applied Chemistry and Environmental Science, RMIT University, Melbourne, 3001 Australia; 7 Department of Physiology, Monash University, Clayton, 3800 Australia; 8 School of Engineering, RMIT University, Melbourne, 3001 Australia; 9 ARC Centre of Excellence for Nanoscale BioPhotonics, School of Science, RMIT University, Melbourne, 3001 Australia

**Keywords:** Colour vision, Plant signalling, Electron microscopy

## Abstract

Plant organs including flowers and leaves typically have a variety of different micro-structures present on the epidermal surface. These structures can produce measurable optical effects with viewing angle including shifts in peak reflectance and intensity; however, these different structures can also modulate hydrophobic properties of the surfaces. For some species optical effects have been proposed to act as signals to enhance pollination interactions, whilst the ability to efficiently shed water provides physiological advantages to plants in terms of gas exchange and reducing infections. Currently, little is known about epidermal surface structure of flowering plants in the Southern Hemisphere, and how micro-surface may be related with either hydrophobicity or visual signalling. We measured four Australian native species and two naturalised species using a combination of techniques including SEM imaging, spectral sampling with a goniometer and contact angle measurements. Spectral data were evaluated in relation to published psychophysics results for important pollinators and reveal that potential visual changes, where present, were unlikely to be perceived by relevant pollinators. Nevertheless, hydrophobicity also did not simply explain petal surfaces as similar structures could in some cases result in very different levels of water repellency.

## Introduction

The variety and complexity in micro-surfaces (also referred to as micro-sculpture) present on the epidermal surface of different plant organs such as leaves and flowers has long been recognised by plant scientists for taxonomic identification^1^. From a sample of about 5,000 plant species including Angiosperms and Gymnosperms, Bartholott identified four main characteristics defining a plant micro-surface: cell morphology, relief of the cell wall, waxes and epicuticular secretions, and multi cellular structures including trichomas (hairs) and glands^1,2^. Much effort has been devoted to sample and characterise these microstructures using different microscopy techniques including scanning electron microscopy (SEM), and transmission electron microscopy (TEM) for taxonomic purposes^3^; however, the underlying cause of such a morphological diversity still remains unclear.

One of the first explanations for the origin and diversity of epidermal micro-surfaces is water repellency^4^. Excess of liquid due to rainfall or dew can be detrimental to the plant as it reduces photosynthetic activity, promotes the development and spread of fungal infections, facilitates pollutant deposition, and accelerates foliage nutrient leaching^4–6^. Considering the negative effects of excess water on plant surface, features that enhance water repellency would be assumed to be favoured over those that do not^4^, and it is expected that different adaptations for water repellency will be present on plants living in places with distinct environmental conditions, particularly in habitats exposed to frequent rainfall or dew^1,5^. Indeed, Christensen and Hansen [3] hypothesised that flat epidermal cells were ancestral traits that subsequently developed into the papillate type frequently observed among Angiosperms.

Hydrophobicity in plants can be attained either by the roughness of a surface which prevents absorption of the liquid, i.e. the Wenzel effect^7^, or by the formation of air pockets between the drop and surface facilitating the rolling of droplets and hence water repellency, i.e. the Cassie and Baxter effect^8^. Both cell morphology and cuticle thickness are related to surface roughness and therefore determine the repellency characteristic of a plant surface^9^. Wettability in various plant organs can be quantitatively assessed through magnitude of the contact angle ($\theta _{c}$) formed between the measured plant’s organ and line tangent to a drop of water standing on its surface^5,10^. If the tissue resists wetting, then the droplet shape will be more curved than the case of an hydrophilic surface with $\theta _{c}<90$ .

The range of contact angles measured on the leaves of several plant species from a wide range of families^2,5,10^, goes from super hydrophobic surfaces ($\theta _{c}\approx 150^{\circ }$) in the sacred lotus (*Nelumbo nucifera*)^11^, to the highly hydrophilic leaves ($\theta _{c}\approx 24^{\circ }$) of *Arnica cordifolia*^5^. Interestingly, large variations in $\theta _{c}$ values are not only observed among species and habitats, but also among plant organs such as leaves and flowers; and, even between the ‘upper’ (adaxial) and ‘lower’ (abaxial) surfaces of the same organ^12^.

Sampling over a wide range of plant species in the Northern Hemisphere indicates that conical or papillate cells are often present in flower petals with only few species presenting tubular cells^3, 9,13,14^. This observation, in addition to the lack of photosynthetic activity in most flower petals, suggests that conical cells may provide advantages to these organs other than water repellency. Alternative roles for micro-surfaces in flowers include providing tactile cues and facilitating flower handling by insect pollinators^15^, and the production of angle-dependent colouration signals for visual communication between plant and insect^13,16,17^.

Flowers in both the Northern^18^ and Southern^19^ Hemispheres have evolved in parallel to best suit the visual capabilities of bees. The appearance of a flower to a pollinator results from interaction between the absorption profiles and concentration of pigments present at its various tissue layers; their number, arrangement and optical properties^14,20–23^. In addition, the morphology of cuticle cells allows for the production of various optical interference effects as incident light interacts with the regular or semi-regular petal features at micro or nano scales. This interaction produces structural colours with different types of visual effects including iridescence^24,25^. In some cases, these colours may contribute to the overall chromatic appearance of the petal which is in most cases dominated by the diffuse coloration produced by pigments distributed across the different tissue layers of the flower^14,20^.

The production of iridescent colours by some flower species such as *Hibiscus trionum* in laboratory conditions has lead to the hypothesis that micro surfaces on petals may have specifically evolved for the production of visual signals to pollinators^17^. However, van der Kooi et al.^26^ showed that if present, iridescent signals would be an unreliable source of information for free flying bees. Indeed, considering plant species from the Southern Hemisphere, including Australia, Garcia et al.^27^, showed using quantitative imaging techniques that flowers from within and outside the order to which *Hibiscus trionum* belongs produce a limited amount of highly variable patches of angle-dependent colours when seen under variable lighting conditions^27^. Moreover, we also showed using psychophysic experiments that systematically controlled for colour information potentially available from different angles of view, that free flying honeybees ignored angle-dependent information when salient, angle independent, pigment based colour was available.

In the current study we consider the same six plant species used in Ref.^27^ to understand if the diversity of micro-surfaces observed on the petal sample may be correlated with the hydrophobic properties of the petals, as predicted by the water repellency adaptation hypothesis. Subsequently, we characterise both the specular and diffuse components of the coloration displayed by the two species at the extremes of our sample hydrophobicity range by recording reflectance spectra at different sensor and light geometries, and use visual models for the honeybee *Apis mellifera*, a model bee pollinator, to test for the potential perception of angle-dependent stimuli. Reflectance measurements allow for the complete characterisation of a structural colour by separately measuring changes of intensity and wavelength of peak reflectance dependent on visual geometry. For a human observer, these changes are perceived as either ‘gloss’ or iridescence, respectively. Finally, we model angular measurements of intensity and maximum wavelength using psychopysics-informed visual models to test if measured changes in spectral profiles are potentially perceivable by a bee observer under an open sky illumination and those typical of forests. By considering different lighting conditions we test for the alternative role of iridescence for increasing flower detection by bee pollinators rather than just improving discrimination as considered by previous studies^27^.

## Results

### SEM imaging and petal cell morphology

**Fig. 1:**
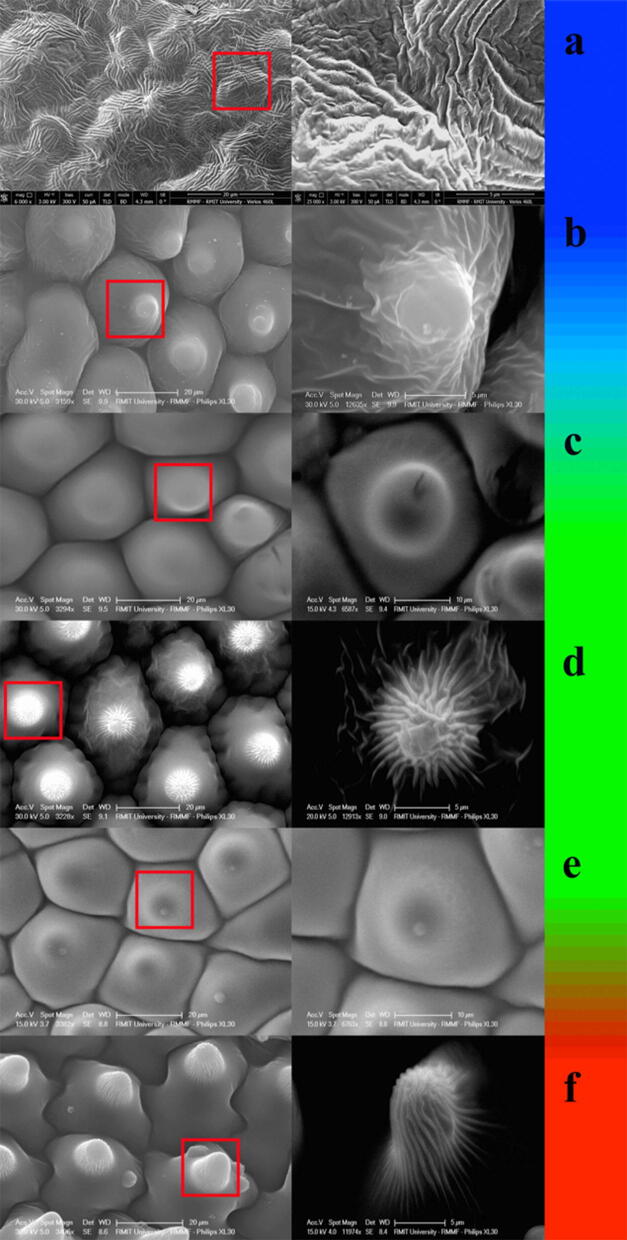
SEM micrographs of petal epidermal micro-surfaces. Colour bar indicates contact angle magnitude sorted in ascending order from the top. Blue colour corresponds to the smallest contact angle whilst red represent the largest value. Left column shows SEM images at low magnification whilst the right column depicts details of cellular features. Magnification settings for each image are presented in Table 1 along with flower sizes. Species are indicated by letters: (**a**) *A. huegelii*, (**b**) *H. heterophyllus*, (**c**) *L. rantonnetii*, (**d**) *P. rodneyanum*, (**e**) *S. laciniatum* and (**f**) *T. majus*. Note how species such as *A. huegelii* (**a**) and *H. heterophyllus* (**b**), although presenting similar contact angles, differ in cell micro-surface. Refer to Table 2 for further details.

Scanning Electron Imaging revealed that the petal surface of our sample contained tabular (flat), convex conical and convex papillate cells. Cuticular foldings were identified in three out of the six sampled species (Fig. 1, Table 2).

**Table 1:** Mean flower size, measured as diameter^45,46^, and magnification settings used to obtain SEM images of petal micro-surface and feature details (in brackets) from the six species discussed in the present study.

Species	Size (mm)	Magnification
*Alyogyne huegelii*	90	$6,000,\,(25,000)\times$
*Hibiscus heterophyllus*	95	$1,580\,(3,160)\times$
*Lycianthes rantonnetii*	36	$832\,(3,290) \times$
*Pelargonium rodneyanum*	32	$1,500\,(12,900)times$
*Solanum laciniatum*	40	$3,380\,(6,760)\times$
*Tropaeolum majus*	75	$3,400\,(12,000)\times$

### Contact angle measurements

**Fig. 2:**
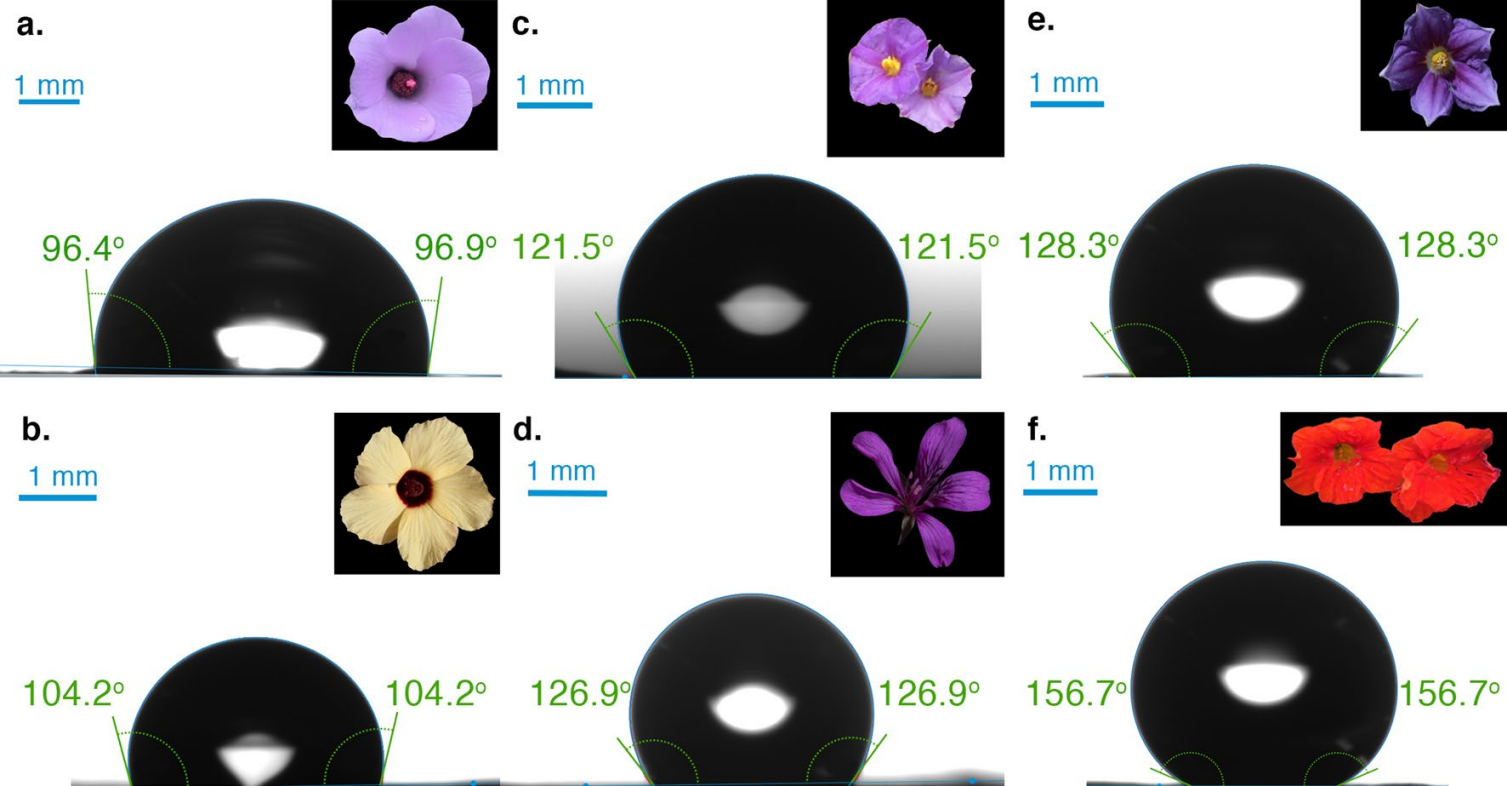
Sample images of the species selected for the study (inserts) and examples of corresponding contact angle measurements for: (**a**) *A. huegelii* (mean contact angle for adaxial petal tip ${\bar{\theta }}_c = 99.5^{\circ }$), (**b**) *H. heterophyllus* (${\bar{\theta }}_c = 101^{\circ }$), (**c**) *L. rantonnetii* (${\bar{\theta }}_c = 120^{\circ }$), (**d**) *P. rodneyanum* (${\bar{\theta }}_c = 130^{\circ }$), (**e**) *S. laciniatum* (${\bar{\theta }}_c = 128^{\circ }$), and (**f**) *T. majus* (mean contact angle for petal tip on the adaxial surface of the lateral petal ${\bar{\theta }}_c = 153^{\circ }$). Milli-Q water was used as test liquid in all cases. Droplet sizes used for measuring contact angle for each species were: $10.7\, \upmu \hbox {l}$ for *A. huegelii*, $2.60\, \upmu \hbox {l}$ for *H. heterophyllus*, $5.39\, \upmu \hbox {l}$ for *L. rantonnetii*, $3.16\, \upmu \hbox {l}$ for *P. rodneyanum*, $9.31\, \upmu \hbox {l}$ for *S. laciniatum*, and $10.3\, \upmu \hbox {l}$ for *T. majus*. Droplet sizes were chosen to ensure they properly rested on flat areas of petals with various sizes, features and morphology. Scale bar represents 1 mm for the droplet image in all cases. See Table 2 for results of all measurements.

We obtained contact angle values on the adaxial (top) surface of the petals ranging from $94.1^{\circ }$ in *A. huegelii* to $153.0^{\circ }$ in *T. majus* (Fig. 2). On the abaxial surface of the petals $\theta _c$ values ranged from $77.5^{\circ }$ in *A. huegelii* to $150.9^{\circ }$ in *T. majus*. Individual $\theta _c$ values for the six samples are provided in Table 2.

**Table 2:**
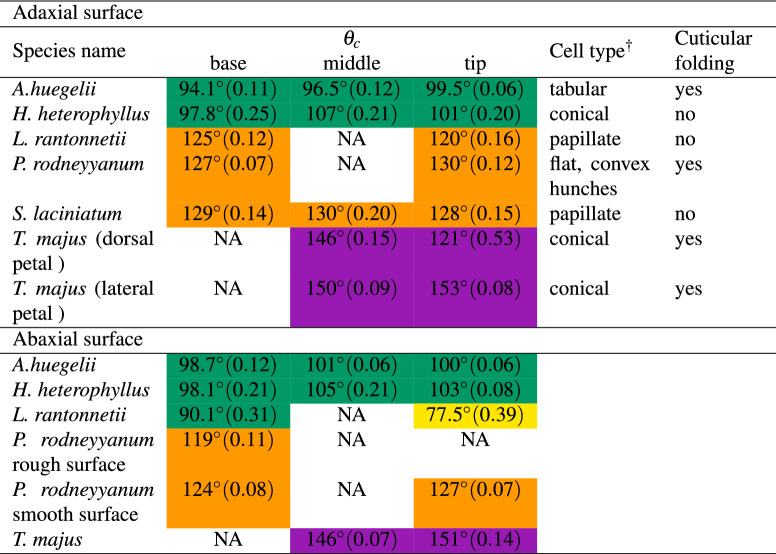
Mean contact angle ($\theta _{c}$) and type of cells present on the adaxial and abaxial surfaces of the six species used in this study.

Mean contact angle values were obtained from three independent measurements recorded on three different regions: tip middle and bottom from petals of two different flowers. Measured $\theta _{c}$ values ranged from wettable ($\theta _{c}< 110^{\circ }$) to highly non-wettable surfaces ($130^{\circ }<\theta _{c}< 150^{\circ }$). Values in parentheses represent the sample circular standard deviation. $^\dagger$ Cell types follow the nomenclature by Koch et al. [2]. Colour codes in table represent the different surface wettability categories defined by Taneda et al. [9]: highly wettable (yellow), wettable (green), non-wettable (orange) and highly non-wettable (pink).

### Spectral measurements

**Fig. 3:**
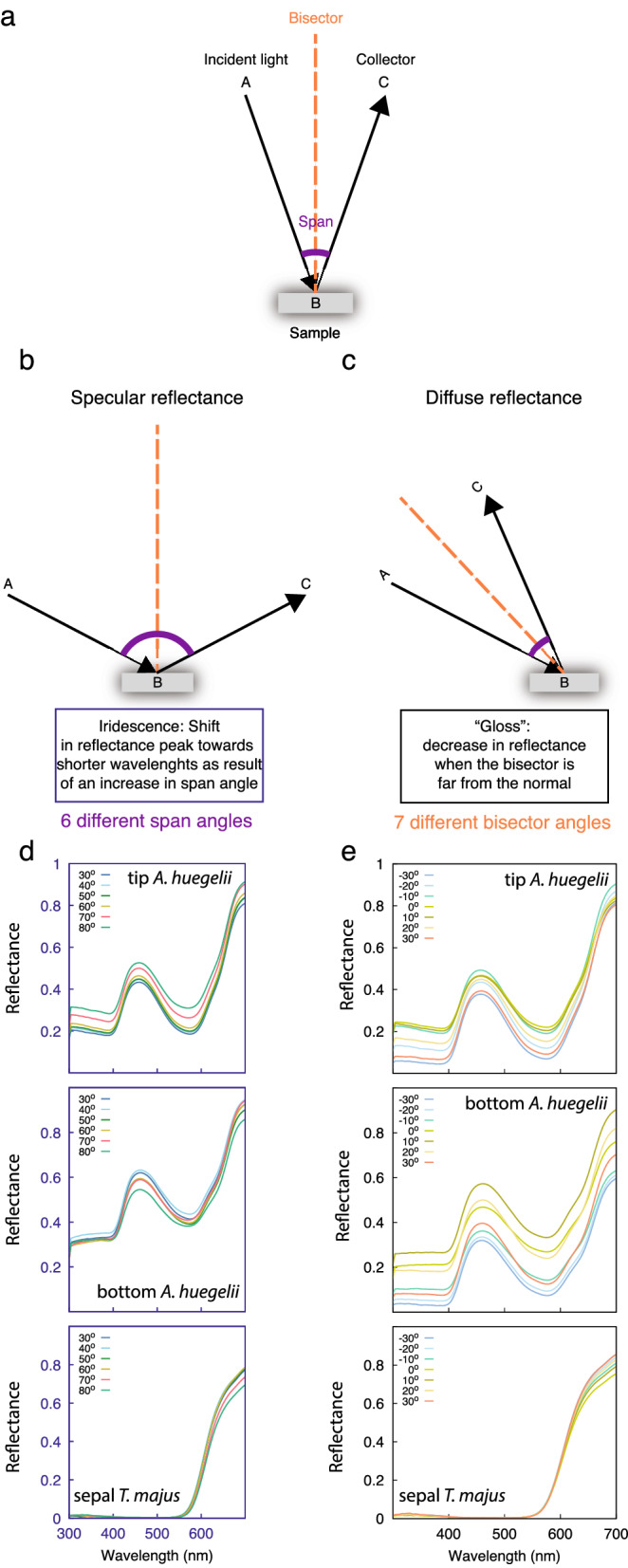
Schematic of the goniometer setup (**a**) employed to measure spectra corresponding to the two main components of a structural colour: iridescence (**b**, **d**) and ‘gloss’ (**c**, **e**). Spectra in panels (**d**) and (**e**) were collected from the tip (top row) and bottom regions (middle row) of *A. huegelii *, and the lateral sepal of *T. majus* (bottom row). Spectra corresponding to the iridescence measurements (**d**) were obtained using span angles of $30^{\circ },\, 40^{\circ },\, 50^{\circ },\, 60^{\circ },\, 70^{\circ }$ and $80^{\circ }$ (**b**) as indicated in legend for panel (**d**). Spectra measuring changes in ‘gloss’ (**e**) were recorded by positioning the bisector $-30^{\circ },\, -20^{\circ },\, -10^{\circ },\, 0^{\circ },\, 10^{\circ },\, 20^{\circ }$ and $30^{\circ }$ from the Normal (**c**) as indicated by the legend. Refer to the spectral measurements subsection under Methods for details.

Spectra from *A. huegelii* and *T. majus*, representing the extremes of the range of contact angle measured, i.e the most hydrophilic and hydrophobic species, were obtained at various span and bisector combinations to test for potential changes in appearance with viewing angle (Fig. 3). Due to the differences in hydrophobicity observed between the tip and bottom of *A. huegelii*, we measured these two regions independently for this species. Measurements obtained from various span angle combinations with a fixed bisector indicate that the two species are very unlikely to display the characteristic blue shifting in $\lambda _{\max }$ associated with higher span, which would be considered as iridescence. Moreover, peak reflectance was found to be independent from span angle in all tested cases: Mardia rank correlation coefficient (*U*) for *A. huegelii*: $U_{A. huegelii}\,tip = 3.00,\,P=0.900;\,U_{A. huegelii}\,bottom = 3.00,\,P=0.908;\,U_{T. majus}=12.0,\,P=0.694$. *T. majus* maintained $\lambda _{\max }=650$ nm across all measured span angles.

**Fig. 4:**
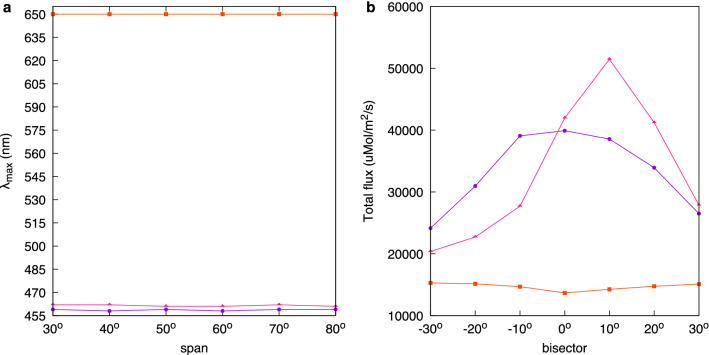
Colour and intensity changes as a function of viewing angle for the tip (purple lines) and bottom regions (pink line) of *A. huegelii * and the lateral sepal of *T. majus* (red line). (**a**) Spectral position of the reflectance peak ($\lambda _{\max }$) of measurements collected at various span angles whilst keeping the bisector angle fixed. We did not observe changes in the position of $\lambda _{max}$ with varying span in the three samples tested suggesting a lack of iridescence in both *A. huegelii* and *T. majus*. (**b**) Total intensity for the reflectance measurements obtained for each sample from readings at different bisector angles whilst holding the span angle fixed. We observed changes in total intensity with bisector angle for the tip and bottom petal regions of *A. huegelii*, consistent with the perception of ‘gloss’ with viewing angle by a human observer.

The two petal regions of *A. huegelii* differed in the position of their mean peak wavelength (± standard deviation from the mean): ${\lambda _{\max }}_{tip}=459\,(\pm 0.516)$ nm, ${\lambda _{\max }}_{bottom}=462\,(\pm 0.548)$ nm. There was a subtle change in $\lambda _{\max }$ by 1 nm across the measured span angles (panel a, Fig. 4); however, this difference is not larger than variation resulting from mathematical operations on the raw signal implemented during data processing such as smoothing.

Potential changes in total reflectance from the three samples were obtained from measurements with a fixed span and changing the bisector angle. There was a significant correlation between bisector angle and total intensity for the three samples of *A. huegelii*: $U_{A. huegelii}\,bottom=92.2,\,P=0.018;\,U_{A. huegelii}\,tip=92.5,\,P=0.008;\,U_{T.majus}=92.5,\,P=0.014$.

**Fig. 5:**
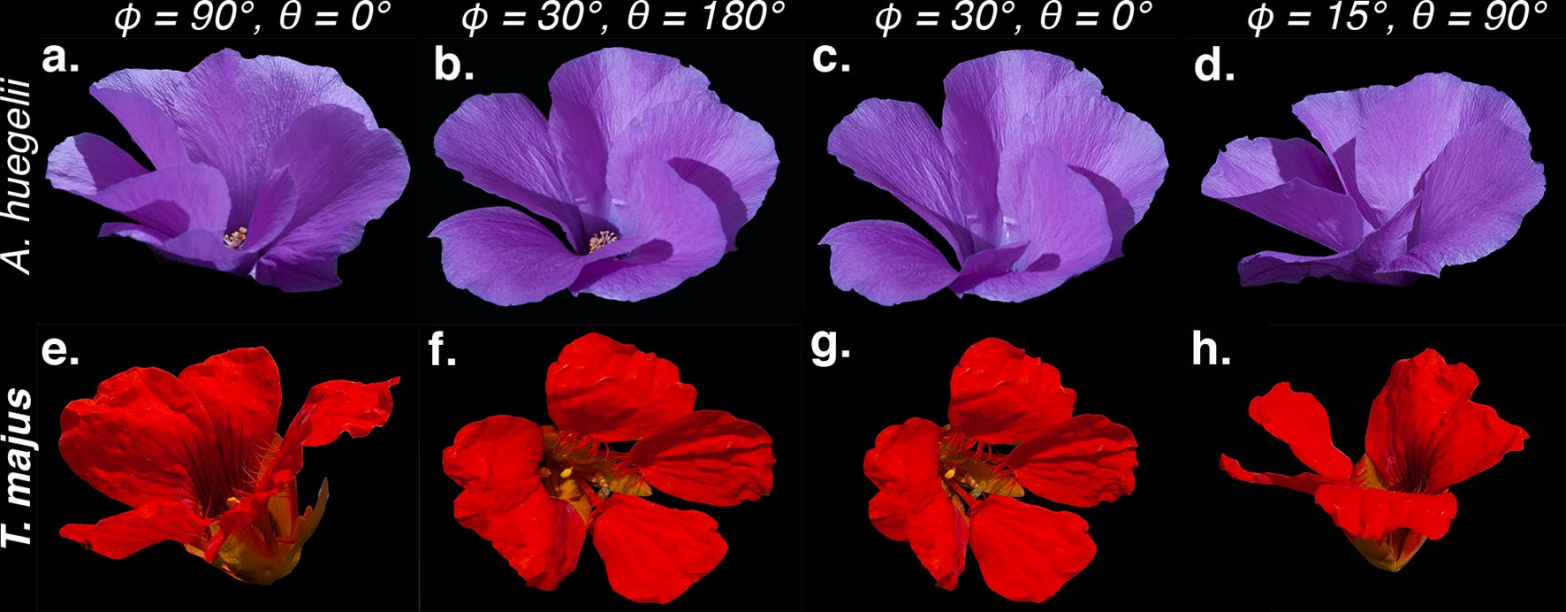
RGB colour images of *A. huegelii* (**a**–**d**, upper row) and *T. majus* (**e**–**h**, bottom row) obtained at different inclination ($\phi$) and orientation ($\theta$) angles for the same specimen. Note how the bottom and tip regions on the petal of *A. huegelii* display a distinctive ‘gloss’ at some viewpoints (**a**, **c**), whilst petals of *T. majus* display a matte coloration across all viewpoints consistent with measured changes in total intensity for the two species (Fig. 4). Refer to^27^ for details on the photographic method used for obtaining the images.

When considering a typical, open sky illumination, changes in reflected intensity for *A. huegelii* were highest for the bottom region of the petal with an 2.5 times increase within the range of measured bisector angles ($20.368\, \upmu {\hbox {mol/m}}^2/\hbox {s} ,\,51.456\, \upmu {\hbox {mol/m}}^2/\hbox {s}$), followed by the tip region of the same species with a maximum change of about 1.6 times in the amount of reflected flux ($24.136\, \upmu {\hbox {mol/m}}^2/\hbox {s} ,\,39.903\, \upmu {\hbox {mol/m}}^2/\hbox {s}$). For *T. majus* we did not observe a change in reflected flux higher than 0.1 times ($13.679\, \upmu {\hbox {mol/m}}^2/\hbox {s} ,\,15.301\, \upmu {\hbox {mol/m}}^2/\hbox {s}$) (**b**, Fig. 4). For a human eye, the changes in total intensity of *A. huegelii* are perceived as an angle-dependent, ‘gloss’ effect (Fig. 5, panels a–d). On the other hand, changes in the total flux of for *T. majus* was not higher than 10%, thus petals of this species display a matte, i.e. diffuse, coloration across the measured range (Fig. 5, panels e–h).

### Visual modelling

To serve as visual signals, angle-dependent colours produced by a flower should be readily perceived by its target pollinators. This implies that the observer should be able to discriminate between two spectral signals differing in the position of their peak reflectance. For honeybees this relationship is modelled by the delta lambda/lambda function ($\Delta \lambda /\lambda$) which predicts changes in the probability of discriminating coloured stimuli based on the difference between peak reflectance values of various quasi-monochromatic stimuli.

Reflectance measurements collected with a fixed bisector (Fig. 3) indicate a shift of $\lambda _{\max }$ in both the tip and bottom regions of *A. huegelii* of about 1 nm along the measured span angles ($\Delta \lambda _{\max } = 1$ nm), whilst no change in $\lambda _{\max }$ was observed for *T. majus* ($\Delta \lambda _{\max } = 0$ nm). Such changes in $\lambda _{\max }$ are below the minimum discrimination threshold value of $\Delta \lambda _{\max } = 4.5$  nm at 390 nm for honeybees as determined by psychophysics experiments^28^, although humans can discriminate up to 1 nm differences under optimal conditions^29^.

**Fig. 6:**
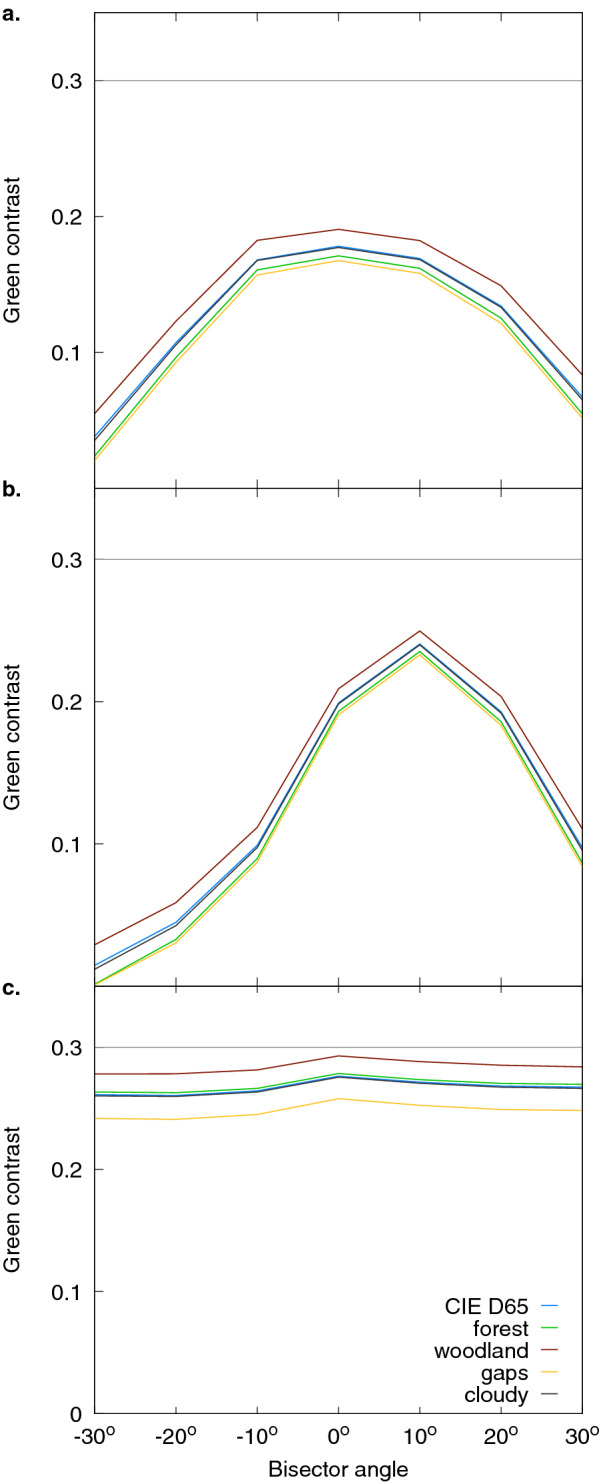
Green contrast calculated from reflectance spectra obtained at different bisector angles for the tip and bottom petal regions of *A. huegelii* (**a** and **b**, respectively) and the lateral sepal of *T. majus* (**c**), assuming different illuminations: open sky during midday in the northern hemisphere, CIE D65 (blue line), forest and woodland shade (green and brown lines), small gaps (yellow line) and cloudy condition (black line). Dashed grey line represent the green contrast threshold value required for detection of small targets by honeybees [30] (see text for details). As green contrast values for our samples are always below the detection threshold for all illumination conditions considered, it may be concluded that changes in total intensity for our samples are not likely to be perceivable by honeybees under most common natural ambient illuminations.

Green contrast values calculated from the reflectance readings at various bisector settings with a fixed span are provided for the five illumination conditions considered are presented in Fig. 6. One way ANOVAs performed on the green contrast values for the tip and bottom petal regions of *A. huegelii* and the lateral sepal of *T. majus* obtained under the five illuminations considered, revealed significant differences between at least two of the illumination conditions considered for *T. majus* ($F = 36.1,\; df=4,\,30\;P<0.001$) but failed to reject the null hypothesis of equality for both the tip and bottom regions of *A. huegelii* ($F_{tip}=0.268,\,df_{tip}=4,\,30,\;P=0.896$, $F_{bottom}=0.080,\,df_{tip}=4,\,30,\;P=0.988$). However, all of the green contrast values were lower than the 0.3 threshold value that honeybees have been shown not to be able to detect in free-flying psychophysics experiments^30^, thus suggesting that bees can not process this visual information in the achromatic sensory dimension.

## Discussion

Measured contact angle and SEM images from the micro-surfaces of our sample indicate that cell morphology is not a unequivocal predictor of the water repellency properties of a flower. For example, even though petals from both *H. heterophyllus* and *T. majus* present conical cells with cuticular foldings (striations) on their surfaces (Fig. 1), their water repellency properties are strikingly different with one species being wettable whilst the other one is highly non-wettable (Table 2). Likewise, species with different cell types can have comparable contact angles such as the case of *L. rantonnetii* and *P. rodneyanum* (Table 2).

The complex relationship between morphology and water repellency properties may also be responsible for the inconclusive support from surveys looking for a direct relationship between water repellency properties of plants and humidity in their habitats. For example, Curtis et al.^31^ did not find evidence supporting the hypothesis of differences in water repellency properties in plants inhabiting tropical montane cloud forests, tropical dry forests and semiarid grassland-foothills ecotones. Likewise Taneda et al.^9^, did not find evidence for such a relationship when considering seven species from the alpine regions of eastern Nepalese Himalayas. Similarly, in our sample from native Australian flowers we observed species adapted to warm, moist tropical environments such as *H. heterophyllus* having comparable contact angles to those of *A. huegelli*, a species mainly growing on subtropical sandy coastal shrub land and heathlands (Table 2). Nevertheless, when we compare larger geographical scales as for example the South American *T. majus* with the Australian *A. huegelli*, we found that there is a large difference in the magnitude of contact angle between tropical and Australian species (Table 2). Interestingly, the two South American species: *L. laciniatum* and *T. majus* have both non-wettable petals.

Another important aspect to consider when measuring the hydrophobic properties of plant surfaces and their potential adaptive benefits is flowering time. For example two of the wettable flowers in our sample: *A. huegelli* and *H. heterophyllus* have very short flowering times within a range of 3–6 h in a single day (M.S personal observation). On the other hand, flowers such as *L. laciniatum* and *P. rodneyanum* that open up to four days have higher contact angles. This suggests the possibility that water repellency constitutes an advantage for reducing the detrimental effect of moist and water over longer time periods^6,32^. Thus temporal dynamics of flowering may be an important factor to consider for future work.

It is also possible that other aspects such as pollinator attraction, in particular to insects, are responsible for the hydrophobic properties observed in our sample. For example, honeybees are know to learn and discriminate between different micro-surfaces of flower petals^15^ and bumblebees can use tactile cues in addition to colour information for discriminating between stimuli^33^. However, it is the role of petal micro surfaces on shaping the visual appearance of flowers which has received more attention in recent years^13,25^.

Whilst the role of different pigments present on a petal’s epidermal layers on the chromatic appearance of flowers are beginning to be better understood thanks to the use of multi-layer optical models^20–23,34^, the hypothesis of evolution micro-surfaces for producing visual signals through iridescence by flowers remains a matter of debate as there is conflicting evidence on whether and in which contexts structural colours do serve as signals for visual communication between plant and insects. From the classic definition of signal in the context of animal communication^35^, any colour displayed by a flower, iridescent or diffuse, should be detected and processed by the animal sensory system and brain to drive a decision in the receiver^27,35^. After independently modelling two important components of a structural colour; namely changes in the position of peak reflectance, loosely associated with ‘hue’, and total intensity, we could not find evidence supporting the role of angle-dependent colours for visual communication between plant and bee pollinator even when considering the potential effect of different ambient illumination conditions. Although our modelling shows that some optical effects can be more pronounced when produced under certain natural illumination conditions that may be detected by scientific apparatus or a human observer (Fig. 6), such changes are not necessarily perceived for an insect pollinator. Indeed, the presence of even salient colours from optical effects, like a rainbow^36^, cannot be assumed to be visual signals. More specifically, Fig. 6 shows that the green contrast values calculated from the different spectral measurements and illuminations were never equal or higher than the $G_c=0.3$ value required to be exceeded for target detection by honeybees as validated by recent behavioural studies^30^. Similarly, changes in the position of peak reflectance with angle, ($\Delta \lambda$) were always found to be less than 1 nm. Such as difference is very unlikely to be discriminable by honeybees as established by psychophysic experiments^28^.

Blue shifting of the peak reflectance wavelength $\lambda _{\max }$, commonly associated with iridescence, has been observed in some flower species either under lab conditions^24^, when illuminating single petal cells^26^, or from artificial models of petals^25^. However, such changes were not observed in our samples (Fig. 3). If complex, papillated micro surfaces enable the production of iridescent colours which are advantageous for attracting pollinators^13^ or to increase flower detectability^37^, we would expect that species with complex micro-surfaces, thus the more hydrophobic such as *T. majus* (Fig. 1), are more likely to show the blue shift in $\lambda _{\max }$ typical of iridescence. We did not observe this phenomena even after considering different types of natural illumination corresponding to a wide variety of forest environments. Indeed, the most hydrophobic flower in our sample (*T. majus*) presented a matte coloration typical of diffuse pigment colours (Figs. 4, 5). Thus textured micro-surfaces like the one of *T. majus* may serve to diffuse specular reflection; although more data is required to test this theory.

Our results do not discard the possibility that micro-surfaces present on flower surfaces increase hydrophobicity and, under certain specific conditions, can produce structural colours that interact and modify the diffuse pigment coloration as reported for several species^20,21,34^, as highly complex and textured micro-surfaces can introduce irregularities in the surfaces which may enhance the diffuse component of the colouration. However, our results suggest that even if angle-dependent effects such as iridescence can be measured, they are unlikely to be perceivable by bee pollinators under a variety of naturally occurring illuminations. Therefore, structural colours in flowers can be the result of a secondary or incidental byproduct of the particular micro-surface of some species. In this case, the hypothesis supporting evolution of micro-surfaces for the sole purpose of visual signalling has to be rejected in favour of an alternative hypothesis. In addition, future work could seek to find out the mechanisms by which flowers with very similar micro-surfaces in some cases have different wettability (Fig. 1), whilst in other cases different micro-structures enable the same properties.

## Methods

### Sample species and distributions

We used the same plant species used by^27^: *Alyogyne huegelii, Hibiscus heterophyllus, Lycianthes rantonnetii, Pelargonium rodneyanum, Solanum laciniatum* and *Tropaeolum majus*; as quality bee behavioural data already exists for these plants. Four of the sampled species are native to Australia and whilst the remaining two are original to South America, these species have been naturalised in Australia. Plants were chosen based on their widespread success in different regions of Australia, and acceptance as commercially useful plants for applications in community and private gardens.

### Contact angle measurements

We measured the contact angle ($\theta _{c}$) of three different flower petals at the top end (tip), middle and basal (base) regions of each petal on both the ventral (adaxial) and dorsal (abaxial) surfaces. Adaxial surfaces are mostly smooth and hairless (glabrous) while abaxial surfaces are hairy (puberulous surface) and present several veins. Water contact angle on petal surfaces were measured using the sessile drop method following protocols by Aryal and Neuner^38^ and Taneda et al.^9^. Milli-Q water (Merck Millipore, VIC, Australia) droplets with an average of $6.91\, \upmu \hbox {l} \pm 3.65\, \upmu \hbox {l}$ standard deviation, were placed on flower petal surfaces using a PC-controlled dosing system and analysed using a Drop Shape Analyser DSA25 (Kruss GmBH, Hamburg, Germany) at room temperature. Droplet sizes for measuring contact angle of each species were selected such that liquid sat comfortably at the centre of surface being measured, and avoiding petal features such as pollen grains and hairs present in puberulous species.

### Microscopy

We used a Philips XL-30 Scanning Electron Microscope (SEM) operating at $30 \, \hbox {kV}$ acceleration voltage to acquire images from flowers casts at high vacuum pressure. We used a set of various magnification settings ranging from about 830 to 25,000$\times$ for scanning the sample chosen depending on the level of detail required in the micrograph. Usage of different settings allowed us to obtain the best possible general and detailed overview of petal micro-structures varying in size. The specific magnification settings used to sample each one of the species are provided in Table 1. Working distance from the sample to current beam was set at $10 \, \hbox {mm}$ . Before SEM imaging, casts were sputtered coated with gold to prevent charging effects. Images were recorded at RMIT Microscopy and Microanalysis Facility (RMMF), RMIT University, Australia.

Casts from the petal samples were made on dental impression medium of low viscosity (elight HD+, Zhermack, Italy) following protocols by van der Kooi et al.^26^. Petals were peeled off as soon as the impression material had polymerised, leaving a negative image of the surface structures in the impression material. Moulds were subsequently filled with transparent nail polish and set to dry for at least 4–5 min obtaining a positive cast of the petal surface. We compared the quality of the cast visually with the structure of the flower petal surfaces. No differences between the surface structure of the fresh flowers and the casts were observed.

### Spectral measurements

We used spectral sampling methods allowing for the independent and accurate measurement of shifts in $\lambda _{\max }$ and total reflectance intensity based on methods developed by Grouson et al.^39^ to quantitatively characterise iridescent colouration in biological samples.

We recorded reflectance spectra at different geometries by varying the combination of angles between the illumination probe (incident light denoted as inc.) and detector (col.). Considering the illumination point as A, the point where light reaches the surface as B, and the collector location as C, the studied combinations are described by the angle ABC denoted as span and bisector, which cuts the span into two congruent angles (Fig. 3a).

We separately evaluated two different components (Fig. 3b, c) of the sample’s coloration: specular and diffuse reflectance. To evaluate the former, we measured the amount of radiation between 300 and 700 nm reflected in an angle equal to the angle of incidence of light. To this end, we maintained a constant bisector normal to the sample’s surface varying the span by $30^{\circ },\, 40^{\circ },\, 50^{\circ },\, 60^{\circ },\, 70^{\circ }$ and $80^{\circ }$ (Fig. 3b). To measure the diffuse reflectance of petal colour, we measured reflected radiation at angles different from the incident angle by keeping a constant span of $20^{\circ }$, whilst placing the bisector at seven different positions: $-30^{\circ },\, -20^{\circ },\, -10^{\circ },\, 0^{\circ },\, 10^{\circ },\, 20^{\circ }$, and $30^{\circ }$ around the normal (Fig. 3c). Together, these two measurements characterise the different angle-dependent effects of structural colour, thus revealing potential iridescence, shift of $\lambda _{\max }$ towards shorter wavelengths with higher span angles; and ‘gloss’, here described as significant decrease in reflectance when the bisector is far from the normal.

The set-up was calibrated using a perfectly diffuse, Spectralon standard (Ocean Optics, USA). After changing the set-up geometry, we re-calibrated the instrument to correct for changes in position of the intersection point between light and detector on the sample’s surface.

### Visual modelling

Reflectance intensity was calculated by multiplying the spectra by the spectral irradiance of the illumination, expressed in photon flux, and integrating over wavelength. Considered illumination types included: open sky, daylight illumination equivalent to CIE D65^40^, forest, woodland shade, small gaps and cloudy light conditions^41^.

Green contrast values ($G_c$) were calculated from excitation values for the long wavelength sensitive honeybee photoreceptor [*E*(*G*)] from each spectra. Specifically, colour contrast is calculated as the difference in excitation of the green sensitive photoreceptor [*E*(*G*)] relative to the background value of 0.5 ($G_c=\mid 0.5 - E(G) \mid$) as described by Ref.^42^. For our modelling we used photoreceptor sensitivity functions by Peitsch et al.^43^ and assumed as adaptation background the average green leaf proposed by Bukovak et al.^44^ for all our calculations.
